# Potential Antiviral Properties of Industrially Important Marine Algal Polysaccharides and Their Significance in Fighting a Future Viral Pandemic

**DOI:** 10.3390/v13091817

**Published:** 2021-09-13

**Authors:** Renu Geetha Bai, Rando Tuvikene

**Affiliations:** School of Natural Sciences and Health, Tallinn University, Narva mnt 29, 10120 Tallinn, Estonia; rantuv@tlu.ee

**Keywords:** marine algae, algal polysaccharides, pandemic, antiviral medicines, sulfated polysaccharides

## Abstract

Over the decades, the world has witnessed diverse virus associated pandemics. The significant inhibitory effects of marine sulfated polysaccharides against SARS-CoV-2 shows its therapeutic potential in future biomedical applications and drug development. Algal polysaccharides exhibited significant role in antimicrobial, antitumor, antioxidative, antiviral, anticoagulant, antihepatotoxic and immunomodulating activities. Owing to their health benefits, the sulfated polysaccharides from marine algae are a great deal of interest globally. Algal polysaccharides such as agar, alginate, carrageenans, porphyran, fucoidan, laminaran and ulvans are investigated for their nutraceutical potential at different stages of infection processes, structural diversity, complexity and mechanism of action. In this review, we focus on the recent antiviral studies of the marine algae-based polysaccharides and their potential towards antiviral medicines.

## 1. Introduction

Marine biotechnology is a rapidly developing arena which utilizes marine organisms to produce, modify or improve biological resources for industrial, food, medical or environmental applications. Considering the pharmaceutical significance of marine bioactive components, various metabolic products from marine biomasses were investigated for their therapeutic efficiencies [1,2]. Algae, being a large part of marine ecosystem, contributes an inevitable role in food and pharmaceutical industry. Algae is a highly diverse group of organisms ranging from microscopic blue green algae to meters long kelps. Seaweeds have numerous commercial applications in products such as stabilizers, thickeners, emulsifiers, etc. [3,4]. Algae are majorly classified into microalgae and macroalgae, the latter is classified based on their pigments as green algae, red algae and brown algae.

Marine algae are low in calories since the lipid content is low. However, they are rich in carbohydrate content and dietary fibers. Marine algae contain large amounts of polysaccharides, majorly as cell wall structural component and storage carbohydrates. The seaweed polysaccharide concentrations vary from 4–76% of the dry weight depend on the species [5]. Cellulose and hemicellulose are the major cell wall polysaccharides. The characteristics of algal polysaccharides depend on species, vegetative stages and season. Considering the storage polysaccharides, in green algae depending on the species, it has polysaccharides such as sulphated galactans, xylans, ulvans, cellulose and xyloglucan. Whereas, brown algae produce alginates, Laminaran, fucoidan and sargassan. Red algae contain agars, carrageenans, floridean starch, porphyran, and xylans [6,7,8].

### 1.1. Biological Properties of Algal Polysaccharides

**Figure 1:**
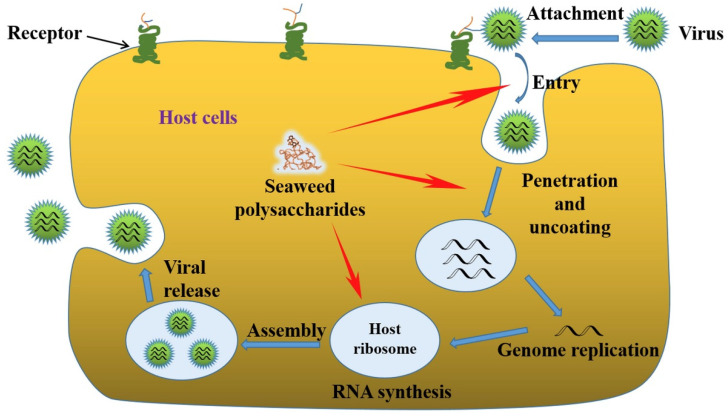
Schematic of viral infection phases and the antiviral actions of seaweed polysaccharides. Reused with permission, license number—5147600647616 [13].

Algae-derived polysaccharides have widely utilized for various biomedical applications considering the pharmaceutical potential, biodegradability and biocompatibility. Algal polysaccharides play vital roles in various bioactivities such as cell–cell interactions, cell additions, and molecular recognition in immune system. The therapeutic efficiency of polysaccharides includes antimicrobial, antifungal, antiviral, antidiabetic, antitumor, antioxidation, anti-inflammatory, immunoregulatory and many more effects [9]. The pharmaceutical uses of polysaccharides depend on the gelation property of the polysaccharide for the preparation of smart drug release carriers. Another factor is the biological response or immunostimulatory properties in terms of antigenicity and immunogenicity for the development of vaccines or drugs [10,11]. However, in presence of an antigen, immunogen initiates an immune response, by triggering the innate immune response and afterward the acquired immune response to alert the body [12]. Herein we discuss more about the antiviral potential of algal polysaccharides. Development of a biocompatible antiviral therapeutic agents from algal polysaccharides must fulfill the criteria regarding safety, biocompatibility, and efficiency. To understand the mode of action and to identify the targets for inhibition, the various life cycle stages of viruses have to be closely considered. The life cycles of viruses are species dependent. As in Figure 1, the six basic stages common for all viruses are attachment to host cells, penetration or entry of virus, uncoating, replication, assembly and release [13].

The antiviral mechanisms of marine polysaccharides involve two major criteria: (1) inhibit the virus activity; and (2) improve the immune response of the host against the virus. As mentioned, virus infection has six basic stages and the antiviral polysaccharides could inhibit viral infection at any of this stage based on their structure, chemical composition and preferred mode of anti-viral action [14]. A direct virucidal action of polysaccharides was observed in many antiviral responses, where the binding of the polysaccharides inactivates the virus itself [15]. Inhibition of viral adsorption is another anti-viral mechanism followed by marine polysaccharides such as galactans, carrageenans, fucoidan and nostoflan. The next mechanism of action is the inhibition of virus internalization and uncoating [16]. The virus internalization is a complex multi-step process including the endocytic uptake, vesicular transport in the cytoplasm, and delivery to endosomes. Another mechanism is the inhibition of virus transcription and replication, which involves direct interference of marine polysaccharides with viral replication enzymes. The alternate method of antiviral action of marine polysaccharides is to improve the host antiviral immune responses. By introducing immunomodulatory marine polysaccharides, the immune response of the host cells is improved by activating production of antiviral immune factors, and thus, indirectly inhibiting the virus replication process and fastening the viral clearance [13].

### 1.2. Significance of Antiviral Activities of Algal Polysaccharides

The recent outbreak of the severe acute respiratory syndrome-related coronavirus 2 (SARS-CoV-2) created a pandemic situation called corona virus disease (COVID-19), which has spread around the globe and is still spreading at an unprecedented rate, creating a serious threat to global public health [17]. Continuous research investigations are happening globally for the development of vaccines and drugs against COVID 19 and the various mutant strains [18,19]. SARS-CoV-virus utilizes host cells’ serine protease TMPRSS2 for the activation of the viral spike (S) glycoproteins by attaching to the host cell receptors ACE2 followed by endocytosis the virus enters the host cells [20]. The SARS-CoV-2 spike glycoprotein (SGP) has major role in the early infection process, where the S1 domain enables the binding and S2 domain mediates the engulfment of the virions by membrane fusion [21]. Glycosaminoglycans (GAGs)—the linear host cell surface protein sulfated polysaccharide, are exploited as attachment factors for host cell entry of SARS-CoV virus, similar to Zika virus [22]. Investigation of the SARS-CoV-2 SGP sequence exposed the furin-like cleavage site at GAG-binding motif resides within S1/S2 proteolytic cleavage motif [23]. Moreover, Kim et al., discovered two additional GAG-binding-like motifs and S20 proteolytic cleavage site in SARS-CoV-2 SGP. When tested, the competitive binding studies of heparin and heparin sulfate with GAG binding motif, heparin sulfate interacts with GAG [24].

Various marine sulfated polysaccharides showed inhibitory activity against SARS-CoV-2, including sea cucumber sulfated polysaccharide (SCSP), fucoidan from brown algae, iota-carrageenan from red algae, and chondroitin sulfate from sharks. Significant antiviral activities are displayed by SCSP, fucoidan, and carrageenan at concentrations of 3.90–500 μg/mL, where SCSP exhibited the best inhibitory activity with IC50 of 9.10 μg/mL. An investigation of pseudotype virus with S glycoprotein confirmed the potential of SCSP to bind to the S glycoprotein to prevent SARS-CoV-2 host cell entry [25]. Additionally, dietary fucoidan is proved as an efficient agent in minimizing pulmonary damage in a model of acute viral infection by enhancing innate immunity and reducing inflammation, thus promoting lung function [26]. Similarly, other sulfated polysaccharides also could be explored for evaluating their antiviral activities.

## 2. Industrially Important Polysaccharides from Marine Algae

### 2.1. Agar

**Figure 2:**
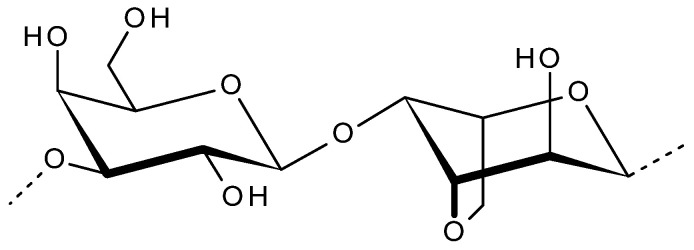
Disaccharide repeating unit of agarose.

Agar is one of the most ancient phycocolloid, whose basic monomer is galactose. It is a hydrophilic gel forming polysaccharide extracted from the cell walls of certain red seaweeds (Rhodophyceae) [27]. The collective term ‘agar’ is used to define a group of polysaccharides made of d and l-galactose [28,29]. The structure of agarose is linear galactan with a backbone comprised of β-d-galactopyranose and 3,6-anhydro-α-l-galactopyranose linked via alternating α-1,3 and β-1,4 glycosidic linkages as shown in Figure 2. Agarobiose and neoagarobiose are the two alternating disaccharide units forming the backbone, where hydroxyl groups are the side chain substitutions [30]. Due to its excellent rheological properties, it has been exploited commercially for applications in food, cosmetic, pharmaceutical, biomedical and biotechnology industries [27]. The major agar-producing red seaweeds (agarophytes) belong to genera Gelidiaceae, Gracilariaceae, Gelidiellaceae and Pterocladiaceae [31,32]. The water temperature, salinity, enzyme activity, nutrients, environment and genetic factors affect the agar content in agarophytes, and thus the yield and gelling properties [28].

The pharmaceutical potential of agar and agarose is explored in various biotechnology applications. Agar is the major component in the culture media for various microbiology experiments. Transparency in sol/gel forms, consistency in gel strength, reliability in gelling/melting temperature, low content of oligomers and proteins, cytocompatibility, and low contamination chances are the key features which enable agar to be an excellent culture media component. In addition to microorganism and cell culture, agar-based biomaterials are also employed in chromatography, electrophoresis, immunology, immobilized enzyme and cell technology [33]. Similarly, agarose microbeads made in combination with polyaldehyde are also utilized in various biomedical applications such as cell labeling, cell separation, affinity chromatography, and hemoperfusion [34]. Furthermore, agarose incorporated biomaterials were extensively employed in wound healing systems and tissue engineering applications [35,36].

*Gracilaria corticate* extract, tested for the antiviral activity against HSV-1 and HSV-2, displayed excellent antiviral potential [37]. A biomarker, named as the ‘d’ character of attenuated strains of poliovirus found unveiled only under agar fluid medium, with low pH and low bicarbonate levels. Agar contained sulphated polysaccharides necessary to the development of the *d* character in the virus. On the contrary, high levels of the sulphated polysaccharide inhibited attenuated (*d*) poliovirus growth. The *d* character thus depends upon the inhibitory effect of the sulphated polysaccharide in the presence of low salt concentrations [38]. Similarly, chicken embryos infected with influenza B virus and mumps virus were treated with *Gelidium cartilagenium* extract containing 0.25% agar, exhibited inhibitory effect on the growth and provided significant protection against the viral infection [39].

In another study, the antiviral potential of agar against dengue-2 virus was evaluated [40]. From the results, the polysaccharide inhibited the virus infection and hemagglutinating properties of dengue virus by direct interaction with the virus particle rather than by interaction with the host cells or with erythrocytes. During the interaction a virus-inhibitor complex was formed with the ratio of virus to inhibitor is approximately 2:1. However, the inhibition of virus activity was reversible by the removal of the agar or by the addition of excess viruses. When inhibitor concentration is high, only a small fraction of the virus is free to contribute to the process of plaque enlargement. Dilution of virus-inhibitor mixtures enables virus recovery. Moreover, studies using other Group B arboviruses specify that these viruses differ in sensitivity to the inhibitor [40]. In another investigation, the dengue Type 2 virus, strain Guinea B, was tested against six different grades of commercial agar in LLC-MK@ cell system. From the observation, the various antiviral effects were observed for different strains of dengue viruses. For dengue Type 1, the plaque development time increased when the agar volume doubled. For dengue Type 4, by keeping agar at 56 °C, plaque was significantly reduced. To compare the antiviral effects of agar, the viruses were cultivated in presence of plaque enhancing compounds and without any chemical additives [41]. When tested against wild type encephalomyocarditis (EMC) virus, a sulfated polysaccharide present in agar successfully inhibited the growth of the minute-plaque former, however, it did not influence the large-plaque mutant. To analyze the role of agar, when tested in the absence of the agar, the minute-plaque former multiplied faster than the mutant [42]. 

To evaluate the direct mode of action of agar inhibitor on Group A arboviruses, the direct interaction between agar polysaccharide and free virus particles are studied. Viruses were propagated in chick fibroblast cells and the inhibitory reactions were observed. The viruses used in the study were Eastern equine encephalitis (EEE) virus, Venezuelan equine encephalitis (VEE) virus and western equine encephalitis (WEE) virus. The inhibition pattern of EEE viruses found similar to dengue-2 and EMC viruses. On contrary, the virus infection rate decreased with incubation time and the agar interaction resulted in reduced plaque counts. Here, the direct inhibitory effect of agar caused hemagglutination. From the results, inhibitory effect is explained as an outcome of the interaction between polysaccharide anionic groups with oppositely charged sites or protein receptors on the viruses. Moreover, the variances in the inhibitory response in different strains of virus could be related to differences in the surface protein charges [43].

In the case of Mengo virus, an agarose overlay yielded faster and bigger plaque formation than any overlays of chemically undefined agars with or without enhancers [44]. However, three different variants (S, M, and L) of Mengo encephalomyelitis virus, were found to be sensitive to the inhibition by agar. The inhibition of the plaque formation is evaluated by agar factor and from the study results displayed that agar factor blocks cell-virus interaction by directly immobilizing the virus particles [45].

### 2.2. Porphyran

**Figure 3:**
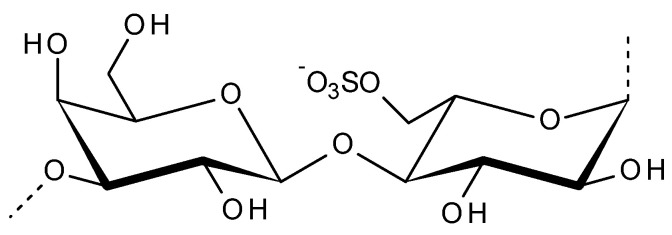
Disaccharide repeating unit of porphyran.

Porphyra and Pyropia species are an important stress tolerant red marine alga, worth ~USD 1.3 billion per year [46]. Sulfated polysaccharides called porphyrans are the main components of Porphyra. Porphyran is a galactose chemically related to agarose, as shown in Figure 3, the structure is highly substituted by the 6-O-sulfation of l-galactose units and 6-O-methylation of d-galactose units [47,48]. Porphyran is one of the most consumed algal products, which is used in food industry as a major ingredient in sushi. *Porphyra yezoensis*, *Porphyra tenera*, *Porphyra haitanensis* and *Porphyra suborbiculata* are traditionally used in Japan as a sheet type of dried food called “Nori”, which contains major dietary fiber and constitutes nearly about 40% of its mass. It is nutritious, having abundant protein, polysaccharide, vitamins, and minerals. Apart from direct uses in food industry, porphyran is also used to analyze the anti-allergic activity of food and food components [49]. The therapeutic potential of porphyrans includes the antioxidant, antitumour, antihyperlipidemic, antifatigue, antibacterial, anticoagulant, anticancer, antiviral, hepatoprotective, hypotensive, and immunomodulatory activities [50]. The antioxidant and anti-inflammatory activities of prophyran from *P. yezoensis* was tested on RAW264.7 cells [51]. 

The anticancer activity of porphyran depends on cell cycle changes and the sequence of apoptotic events resulted from the exposure of porphyran [52]. When tested, porphyran found highly biocompatible with normal cells, where it induced a dose dependent toxicity towards AGS human gastric cancer cells. Introduction of 0.1% porphyran for 24 h of exposure resulted in reduced DNA synthesis, cancer cell growth inhibition, anti-proliferation followed by caspase-3 activation inducing apoptosis [53]. Similarly, when tested on Hep3B, HeLa and MDA-MB-231 cells porphyran showed remarkable anticancer activities where, it had no toxicity in normal human liver cells (HL-7702). Additionally, in the case of HeLa cells, the antitumor activities were related to molecular weight of porphyran and the HeLa cell cycle was blocked in the G2/M phase with antiproliferative effects and altered expression of p21, p53, Cyclin B1 and cyclin-dependent kinase 1 [54].

The study on acid hydrolysis product of porphyran called oligo-porphyran on the glycerol-induced acute renal failure (ARF) model showed it neuroprotective effects in terms of renal morphology and function [55]. *Porphyra umbilicalis* incorporated diets in human papillomavirus Type 16 (HPV16), infected transgenic mice are tested for checking its malignancy development (multistep carcinogenesis) induced by HPV. From the study, the *P. umbilicalis* significantly blocked the development of pre-malignant skin lesions and indicate its antigenotoxic activity against HPV-induced DNA damage [56]. 

Sulfonated-naphthyl porphyran and their copper, iron chelates exhibited antiviral activity against HIV-1. The anti-viral effects on HIV-1 IIIB and HIV-1 JR-FL were tested on epithelial HeLa-CD4-CCR5 cell lines [57]. The antiviral property of porphyran derivatives were tested against HIV-1 using a comparative molecular field analysis (CoMFA) methodology. According to CoMFA, the anionic and hydrophobic groups of porphyran could interact with the highly conserved positively charged and hydrophobic sites, of the V3 loop. This action can induce conformational changes in the gp120 envelope glycoprotein and thus lead to inhibition of virus entry and virus replication [58]. Polysaccharide extracted from *Porphyra haitanensis* in four methods, were utilized in the chick embryo experiments to study the anti-influenza A1 virus activity [59]. The presence of HCV and HIV found efficient in altering the prophyran metabolism and result in photosensitivity which is utilized for the detection of HCV and HIV in blood samples [60].

### 2.3. Carrageenans

**Figure 4:**
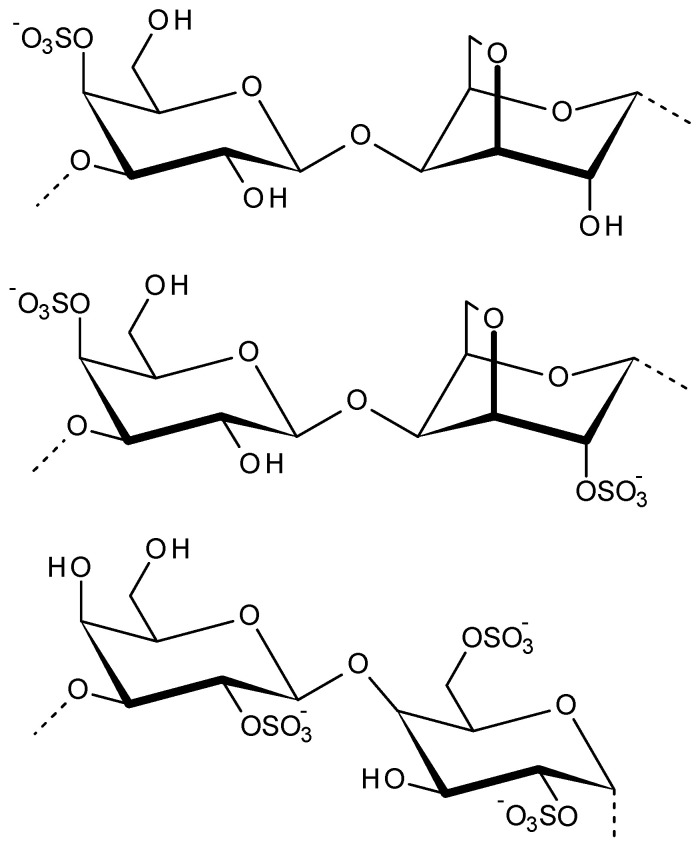
Disaccharide repeating units of kappa, iota and lambda carrageenans (order top to bottom).

Carrageenans are the family of sulfated linear polysaccharides extracted from red seaweeds of main genera like *Chondrus*, *Euchema*, *Furcellaria*, *Gigartina*, *Hypnea*, *Iridae*, *Kappaphycus*, etc. [61]. Approximately 30–75% of the dry weight of these red algal cell walls contains carrageenans. According to their structural characteristics, such as their sulfation patterns and the presence of 3,6-anhydro bridges in α-linked galactose residues, the carrageenans are classified. Carrageenans mainly consist of alternating 3-linked β-d-galactopyranose (G-units) and 4-linked α-d-galactopyranose (D-units) or 4-linked 3,6-anhydro-a-d-galactopyranose (DA-units), as displayed in Figure 4, forming the disaccharide repeating unit of carrageenans [62]. In addition to galactose and sulphate, other carbohydrate residues, such as xylose, glucose and uronic acids, can be present in carrageenans [63]. The various unique physicochemical properties are related to the structural composition of different types of carrageenans. Based on structural variations of carrageenans, the sol-to-gel transition, mechanical strength, chemical crosslinking and biological properties varies. Owing to their unique properties carrageenans are majorly explored in food, cosmetic, printing, textile formulations and pharma industry [64]. These phycocolloid polysaccharides exhibit various biological properties such as anti-thrombotic, anti-viral, anticancer, and immunomodulatory activities. Furthermore, based on the strong negative charge and gelling property, carrageenans are used as a gelling/viscosity enhancing agent for controlled drug release with pH-sensitivity, adhesive property, prolonged retention of drug formulation, tissue regeneration and cell delivery [65,66,67]. Carrageenans strongly bind to metal ions, and thus could be utilized for developing new drugs with high selective metal binding properties. These drugs could be used for elimination of metals from the body or for targeted delivery of these metal ions for healing purposes [68]. 

Carrageenans are originally classified into six basic types and labelled by a Greek prefix based on their chemical characteristics such as: iota (ι)-, kappa (κ)-, lambda (λ)-, mu (μ)-, nu (ν)- and theta (θ)-carrageenan. Apart from this, more varieties of carrageenans are known identified lately. Iota, kappa, and lambda-carrageenan are the main three commercially utilized carrageenans. Each carrageenan is a complex galactose based polysaccharide that has different quantities of sulphate esters at different positions and with different distributions [63]. 

Studies showed that carrageenan has a direct antiviral action on some enveloped viruses, which makes the viruses lose the ability to infect cells, thus effectively reducing the virus multiplication. Various carrageenan formulations reported successful in preventing herpes simplex viruses HSV-1 and HSV-2 [69,70]. The in vitro and in vivo investigations of carrageenans influence on herpes simplex virus (HSV) displayed, virucidal action, virion inactivation, protective effect against virus replication and improved mortality [71,72]. 

Some sexually transmitted human papillomavirus (HPV) types are associated with the development of cervical cancer. Carrageenans prevent the binding of HPV virions to cells. Carrageenans resemble heparan sulfate, an HPV cell-attachment factor, and its HPV inhibition is three times more [73]. In combination with griffithsin—a non-antiretroviral HIV entry inhibitor derived from red algae, carrageenan exhibited excellent biological activity against HSV-2 and HPV in vitro and in vivo [74,75]. 

The antiviral activity of carrageenans was evaluated against HSV-2 and dengue virus DENV-2. The IC50 value of carrageenan against DENV-2 was in range of 0.14–1.6 mg/mL. From the results, compared with *Gymnogongrus griffithsiae* species the extracts and the fraction derived from *Meristiella gelidium* species were more effective inhibitors of DENV-2 [76]. The virucidal activity of carrageenans found to be directly proportional to their molecular weight up to 100 kDa. Carrageenans from red alga *Acanthophora specifira* and brown alga *Hydroclathrus clathratus* were investigated against HSV-1 and Rift valley fever virus (RVFV), where the viral propagation was inhibited by minimal toxicity to the host Vero cells. The viral replication inhibition was by the amplification of P53 gene in HSV-1 and BCL-2 gene in RVFV, respectively [77]. Lambda, kappa, and iota-carrageenans were depolymerized in the presence of ferrous ions or ferrous ions plus ascorbic acid to evaluate the anti-HIV activities in MT-4 cells infected with HTLV-IIIB. Depolymerized kappa- and iota-carrageenan with mean molecular weights of 50 KDa (81–46) exhibited higher anti-HIV activity than other preparations of carrageenans and dextran sulfate [78]. 

The anti-HIV properties of kappa-carrageenan and 3′-azido-3′-deoxythymidine (AZT) conjugates tested on MT-4 cells showed excellent anti-HIV results due to the synergistic antiviral effects [79]. During the 2009 flu pandemic caused by oseltamivir-resistant H1N1 influenza strains, a potent inhibitor of influenza A virus (IAV) iota-carrageenan is tested in vitro. Based on the influenza A nasal spray containing iota-carrageenan for virus infection inhibition was tested on a mouse model with a lethal dose of influenza A PR8/34 H1N1 virus. The post response of the nasal spray in 48 h duration with strong inhibitory results led to a decision for clinical trials in human [80]. In another study, a 2 kDa kappa-carrageenan oligosaccharide, resulted in effectively inhibiting influenza A (H1N1) virus replication in MDCK cells in a dose-dependent manner. The oligosaccharide did not bind to the cell surface of MDCK cells. Contrary to actions of carrageenan polysaccharide, the carrageenan oligosaccharides entered MDCK cells but did not interfere with virus adsorption. Moreover, carrageenan oligosaccharide inhibited mRNA and protein expression of H1N1 virus after its internalization into cells. Additionally, carrageenan oligosaccharide inhibited the virus replication process [81]. A combination drug nasal spray is successfully developed by combining a specific anti-influenza drug Zanamivir along with carrageenan after in vitro and in vivo analysis. This combination found synergistically efficient against different influenza A virus strains such as H1N1, H3N2, H5N1 and H7N7 [82]. The kappa-carrageenan oligosaccharides with a sulphate content of disaccharide (0.8–1.0 mole/mole) and a molecular weight of 1–3 kDa are the most active IAV antiviral agents with excellent inhibitory actions on replication for both in vitro and in vivo [83]. Low molecular weight κ-carrageenans (3, 5, and 10 kDa), acetylated derivatives (acetylation degree of 1.5), acetylated and sulfated derivative of 3 kDa carrageenan (acetylation degree of 1.0 and sulfation degree of 2.4), were investigated towards the anti IAV effects in FM1-induced pulmonary oedema model. Further, 3 kDa kappa-carrageenan with suitable acetylation and sulfation degree was found as a potential IAV inhibitor [84]. When checked the potential to inhibit swine pandemic 2009 H1N1 influenza virus (SW731), kappa-carrageenan significantly inhibit SW731 replication by interfering with adsorption, transcription, and viral protein expression [85].

### 2.4. Alginate

**Figure 5:**
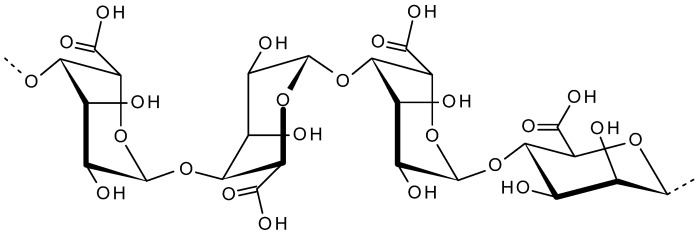
Structure of alginic acid (G and M blocks).

Alginate is a naturally occurring anionic biopolymer from brown seaweed. The major alginate producing brown algae includes *Ascophyllum nodosum*, *Laminaria digitata*, *Laminaria hyperborea*, *Laminaria japonica*, *Macrocystis pyrifera*, etc. [86]. Alginates are linear anionic polysaccharides, and the structures are composed of a main backbone of poly-d-glucuronic acid (G blocks) and poly-d-mannuronic acid (M blocks) as shown in Figure 5. Depending on the M/G ratio, the properties of alginate, such as transmittance, swelling, and viscoelasticity are varied. During the extraction, alginic acid in the brown algae extract is solubilized using an alkaline solution. This alginic acid is treated with sodium salt to obtain sodium alginate, which is the commonly used form of alginate [87]. The proton-catalyzed hydrolysis of alginates dependents on factors such as time, pH, and temperature. Alginate has the unique ability of sol/gel transition, which is it could exist in diverse semisolid or solid structures under specific conditions. Due to this property, alginates are commonly used as emulsion stabilizers, viscosity enhancers, thickeners and suspension stabilizers in food and pharma industry [88]. The structural similarity to extracellular matrices of living tissues allows alginate to be used in wide range of biomedical applications including, pharmaceutical applications, chemical drugs, protein delivery, wound dressing, cell immobilization, encapsulation cell culture, antiviral agents, tissue regeneration with protein and cell delivery [89]. One of the major applications of alginate gels is wound dressings. Alginate dressings can maintain a physiologically moist microenvironment while minimizing bacterial infections and thus facilitates the wound healing process. Being an excellent drug carrier, alginate gels are used for the controlled release of drugs or proteins. Additionally, oral administration of alginate therapeutics is also explored successfully. Alginate biomaterials were extensively explored in the regeneration of skin, bone, cartilage, muscle, nerve, pancreas and liver tissue engineering [88,90]. The antiviral property of alginate is utilized in food industry for the development of novel active edible packaging films along with some lipids, green tea extract and grape seed extract. This alginate biopolymer film exhibited significant antiviral activity against murine norovirus (MNV) and hepatitis A virus (HAV) and thus ensured improved food quality and safety [91].

The antiviral property of alginate gel against hepatitis C virus (HCV) infection in hepatic cell line (HuH-7) was investigated, where the alginate hydrogel protects HuH-7 cells from HCV infection. Moreover, alginate hydrogel also blocked the release of HCV particles from the already infected hydrogel beads and thus prevented further infection. The antiviral interaction was dose and incubation time dependent [92]. An alginate containing polysaccharide drug named 911 displayed potential antiviral activity against AIDS causing HIV-1, for both in vitro and in vivo studies [93]. From the in vitro results, 911 blocked both acute and chronic infection of HIV-1 to MT4 and H9 cells. From the in vivo results, the oral administration of 911 reduced the virus infection and decreased plasma RNA copies. Additionally, introduction of 911 induced a protective effect on CD4 cells, interfered the adsorption of HIV to host cells, significantly suppressed the activity of reverse transcriptase and improved the immune function by antibody development process. Furthermore, the HIV-1 inhibition actions of 911 showed a dose dependent response with low toxicity [93,94,95]. Similarly, to check the anti-viral effect of 911 on hepatitis B virus (HBV) in Hep G2 cells different doses of 911 were introduced. The drug 911 efficiently inhibited the replication of HBV virus by blocking the DNA replication [96].

A dendritic lipid-polymer hybrid nano drug delivery system was made of hybrid nanoparticles of alginate and stearic acid-poly ethylene glycol for the delivery of an anti-HIV drug zidovudine, where the anti-HIV features of alginate could also be explored [97]. Similarly, numerous antiviral drug delivery systems utilize alginate as a perfect biopolymer for the anti-HIV drug delivery vectors [98,99,100]. The efficacy of anti-dengue viral activity of alginate is explored for developing a quantum dot associated quick and efficient virus detection biosensor [101]. Similarly, to prevent the dengue outbreak, calcium alginate-chitosan based microcapsules were designed for the controlled release of imidacloprid larvacide against *Aedes aegypti* larvae and evaluated in both in vitro and in vivo conditions [102].

Sodium alginate microsphere encapsulated plasmid DNA vaccine is orally administered against fish lymphocystis disease virus (LCDV) for immunizing fish in intensive culture due to its ease of operation, low cost and significant immune effect [103]. To formulate a dental impression material with virucidal properties against herpes simplex virus Type 1 (HSV-1), an alginate incorporated biomaterial was compared with eight commercial biomaterials, considering the effect on pH profiles during setting. From the investigation, alginate dental impression materials with pH values of approximately 4.2–4.4 were found to be most efficient in preventing HSV-1 infection [104]. Likewise, for inhibiting HSV-1 infection and arresting the tumor growth, a high mannuronic acid activated alginate macrophages were utilized [105].

### 2.5. Fucoidan

**Figure 6:**
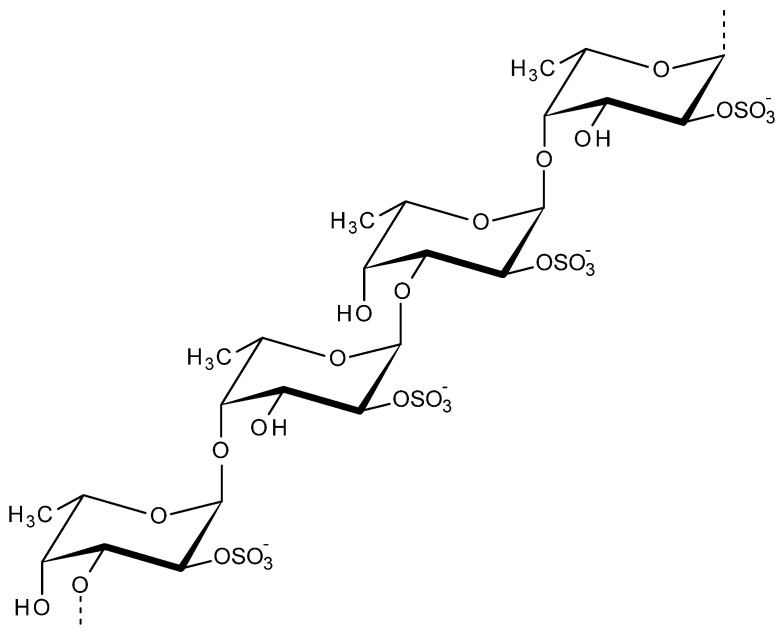
Structure of fucoidan.

Fucoidan is a fucose containing sulfated polysaccharide majorly found in cell–wall matrix of brown algae and in various other marine organisms (certain species of mollusks, echinoderms, arthropods). The major core backbone of fucoidans includes mainly (1-3)-linked α-l-fucopyranosyl structure, additionally, the structure consists of alternating α-(1-3) and α-(1-4)-linked l-fucopyranosyls as shown in Figure 6. Fucoidans could be utilized in food, cosmeceutical, nutraceutical, and pharmaceutical applications. The bioactive properties of fucoidan includes their excellent antibacterial, anticoagulant, anti-inflammatory, antiviral, antithrombosis, anti-tumor, anticancer properties [106,107,108]. Apart from bioactivity, these polysaccharides are associated with future drug development in conjugation with different bioactive enzymes. The most critical issue associated with fucoidan is their large size and season dependent changes in chemical composition [109]. Interesting features like oxidative stress protection and vascular endothelial growth factor (VEGF) interference makes the role of fucoidans critical in ophthalmology [110]. The radioprotective effects of fucoidan was explored via various in vitro and in vivo studies on bone marrow cells to evaluate the cell survival and the immunoreactivity for the utilization in radiotherapy [111]. Fucoidan reported to induce various immune responses. The immunomodulatory activity of fucoidan in association with Jun N-terminal kinase (JNK), nuclear factor kappa B (NF-κB) and nuclear factor of activated T-cells (NFAT) signal pathways are explored in the in vitro, in vivo immunological defense studies [112]. 

Fucoidan exhibited antiviral activity against various RNA and DNA viruses such as HIV, HSV1-2, ASFV, HTLV-1, MPMV, dengue virus, cytomegalovirus, etc. [113,114,115,116]. The antiviral activity of sulfated polysaccharides like fucoidan, dextran/dextrin sulfates depends on its interaction with target cells to inhibit the virus infection. The direct neutralization of virions is not effective. When tested against HIV, HTLV-1 viruses with CD4 receptors, the virus replication was prevented by the binding of fucoidan to the CD4 glycoprotein of the host cells before the internalization process. However, when tested in CD4 deficient host cells like simian retrovirus, MPMV antiviral effects were observed due to the strong binding of fucoidan to an 18 kD membrane protein on T lymphocytes. Fucoidans having higher sulfate content, have capacity to inhibit digestive enzymes like α-glucosidase and α-amylase, thus interrupting or delaying glucose absorption. Similarly, fucoidan influences the enzymes regulating mitosis or cellular apoptosis [115]. 

Antiviral activity of fucoidan extracted from *Sargassum swartzii* has been investigated against HIV. Three bioactive fucoidan fractions were tested and one fraction successfully exhibited 95.6% inhibition in the HIV-1 P24 assay. Moreover, the antiviral activity of the fucoidan fraction was comparable with the standard antiviral drug, with remarkable alterations in the HIV-1 p24 antigen levels and reverse transcriptase activity [117]. Similarly, fucoidans from *Sargassum trichophyllum* also showed antiviral effects towards herpes virus (HSV-2) whereas, fucoidans from *Dictyota dichotoma* exhibited moderate inhibitory effect against herpes virus (HSV-1) and the Coxsackie virus (CVB3) [118,119]. Two types of fucoidans (SHAP-1 and SHAP-2) from *Sargassum henslowianum* exhibited antiviral effects towards HSV-1 and HSV-2. The IC50 values of against HSV-2 were estimated as 0.48 μg/mL by plaque reduction assay. The antiviral activity of fucoidan was via blocking HSV-2 virion adsorption to host cells by preventing its entry to them [120]. Brown algae derived sulfated fucans (from *Dictyota mertensii*, *Fucus vesiculosus*, *Lobophora variegate* and *Spatoglossum schroederi*) were found to inhibit the activity of HIV reverse transcriptase (RT) [121]. 

Native and enzyme modified fucoidans from brown algae *Fucus evanescens* was tested, for the antiviral potential against both DNA and RNA viruses. The antiviral tests were performed on HIV-1, HSV-1, HSV-2 and enterovirus (ECHO-1) both in vitro (Vero and human MT-4 cell lines) and in vivo mice models. In this investigation, compared to the antiviral drug Ribavirin^®^, fucoidans showed multilevel mechanisms such as preventive effect to resist the virus infection, virucidal effect to directly attack the viruses and virus inhibiting effect to inhibit the early-stage virus replication. When comparing the antiviral mechanism, the fucoidans more efficiently inhibited the replication of the DNA-viruses HSV-1 and HSV-2, compared with a moderate antiviral effect against RNA viruses ECHO-1 and HIV-1. From the results, fucoidans affected different stages of HSV-1 and HSV-2 replication more effectively at the stage of virus adsorption and penetration to the host cells. Nevertheless, In the case of HSV, the unmodified fucoidan showed improved antiviral effects, however, in the case of HIV and ECHO, both fucoidans displayed similar antiviral potential [122]. Another fucoidan extracted from sporophytic form of brown algae *Undaria pinnatifida* (Mekabu) was tested against HSV-1, HSV-2, poliovirus and coxsackievirus, HCMV and influenza A virus. The in vitro results exhibited potential dose dependent antiviral effects of Mekabu fucoidan on HSV-1, HSV-2 and human cytomegalovirus for the plaque yield reduction assay to assess the antiviral activity [123]. Following this, the in vivo studies of fucoidan on HSV-1 displayed both humoral and cell-mediated immune responses [124]. 

When tested on avian influenza A viruses (H5N3 and H7N2 subtypes); Mekabu fucoidan was found to be effective in reducing viral replication, suppressing virus yields and increasing antibody production. Additionally, introduction of fucoidan improved the production of neutralizing antibodies and mucosal IgA in the virus inoculated animals as a step towards prevention of viral infection [125]. Similarly, fucoidan when orally administrated on immunocompetent and immunocompromised mice models infected with influenza A virus, the multifaceted antiviral actions involved inhibition to the viral replication, decrease in weight loss and mortality, and extended survival. Oral administration of fucoidan increased neutralizing antibody production in the mice models. Moreover, when treated with oseltamivir, drug resistant viruses were observed after the treatment, however, after Mekabu fucoidan treatment, no drug resistant viruses were found in the mice models [126]. 

### 2.6. Ulvan

**Figure 7:**
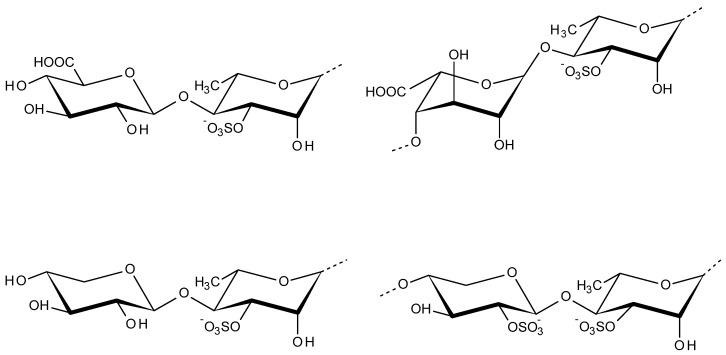
Disaccharide repeating units of ulvan: ulvanobiuronic acids (top left and right units) and ulvanobioses (bottom left and right units).

Ulvales are very common green seaweeds distributed around the globe. Ulvan is the major water-soluble acidic polysaccharide extracted from the cell walls of Ulvales, which accounts for 8−29% of the algal dry weight [127]. Ulvan structure contains disaccharide repeating sequences of sulfated rhamnose and glucuronic acid, iduronic acid, or xylose. The major repeating disaccharides of ulvan are aldobiuronic acids displayed in Figure 7 as ulvanobiuronic acids and ulvanobioses [128]. The morphology of ulvan changes according to pH and salt concentration. At acidic pH conditions, ulvan appeared as dispersed beads whereas, in alkaline pH 13, ulvan forms an open gel-like structure or a continuous film or bead-like structures [129]. Ulvan has the potential to activate plant immunity through jasmonic acid signaling pathway against necrotrophic pathogens as a plant protectant [130]. Ulvans and chemically sulfated derivatives found to be very efficient against phytopathogenic fungi *Alternaria brassicicola* and *Colletotrichum higginsianum* by providing resistance against both fungi, independent of its sulfation degree [131].

The physiochemical and biological properties of ulvan depends on multiple factors such as degree of sulfation, seaweeds species, growth conditions, postharvest stabilization treatment, season, and extraction procedures. Considering the various biological and medicinal properties, ulvan is extensively used in food, pharmacy, agriculture and cosmetics industry. The main biological activities of ulvan include anticancer/anti-proliferative, antioxidant, antihyperlipidemic, anti-inflammatory, anticoagulant, immunostimulatory and antiviral properties. For various biomedical applications including tissue engineering and regenerative medicines, ulvan based materials are used as nanofibers, membranes, particles, hydrogels, 3D porous scaffolds etc [132,133,134]. The innate immunity involves the body’s response to invasive pathogens, inflammatory response, clearance and activation of appropriate acquired responses. Toll-like receptor (TLR) ligands are potent immune-modulators of adaptive immune responses and TLR-based vaccines are broadly explored against infections in chickens. Ulvan extract from *Ulva armoricana* can activate avian heterophils and monocytes in vitro. The activation of heterophils and monocytes induce release of pro-inflammatory cytokines, such as interleukin-1β, interferon α and interferon γ and thus improves the chicken innate immune system [135]. 

To test the biological activity of ulvan from *Ulva pertusa* against vesicular stomatitis virus (VSV), three low molecular weight ulvans were prepared by enzymatic (lyase) degradation and ultrafiltration, such as ulvan-F1, ulvan-F2, and ulvan-F3 with molecular weight 38.5 kDa, 17.8 kDa, and 5.2 kDa, respectively. The antiviral activities of ulvans were evaluated, where they had no cytotoxicity to the Vero cells. However, 100 μg/mL ulvan-F0 and ulvan-F1 showed antiviral activity by inhibiting the infection and replication of VSV. A direct relation was observed with the antiviral activity and molecular weight of the fractions. Ulvan bioactivities could be affected by its molecular weight [136]. 

*Ulvaceae* polysaccharide from *Enteromorpha compressa* was tested against HSV. A sulfated Ulvan fraction labelled SU1F1 was used for testing the antiviral potential against HSV on HEp-2 cells and it is resulted in relevant antiviral activity by SU1F1, with a low IC50 (28.25 µg/mL). The antiviral property is directly related to the sulfation content and molecular weight. Thus, the antiviral effect of SU1F1 until 8 h after infection at high concentrations, suggests the inhibition of DNA replication and transcription with downregulation of HSV protein synthesis [137]. Ulvans from *Ulva clathrata* and their mixture with fucoidans was investigated for another study against paramyxovirus infection as Newcastle disease virus (NDV) in poultry. The in vitro antiviral activity was tested using syncytia reduction assays with monolayers of Vero cells. Ulvans exhibit an IC_50_ of 0.1 μg/mL with a better anti cell-cell spread effect, and inhibited cell-cell fusion via a direct effect on the F0 protein. From the observations, ulvan inhibits syncytia formation only before F protein cleavage. Therefore, the antiviral action of ulvan is by interacting with the intact F0 protein but not with the cleaved mature F protein. Ulvan in combination with fucoidan showed better viral inhibition when tested [138].

Sulfated polysaccharide extracts from *Ulva lactuca* were tested against Japanese encephalitis virus (JEV) infection in Vero cells. JEV is a neurotropic flavivirus causing acute encephalitis in humans. From the response, polysaccharide extracts containing ulvan blocked the virus adsorption and inhibited the virus entry to the host cells. Furthermore, they effectively decreased the production of pro-inflammatory cytokines. From the in vivo studies in C3H/HeN mice models, the JEV-infected mice when treated with ulvan showed improved mortality and delayed infection rates [139]. 

### 2.7. Laminaran

**Figure 8:**
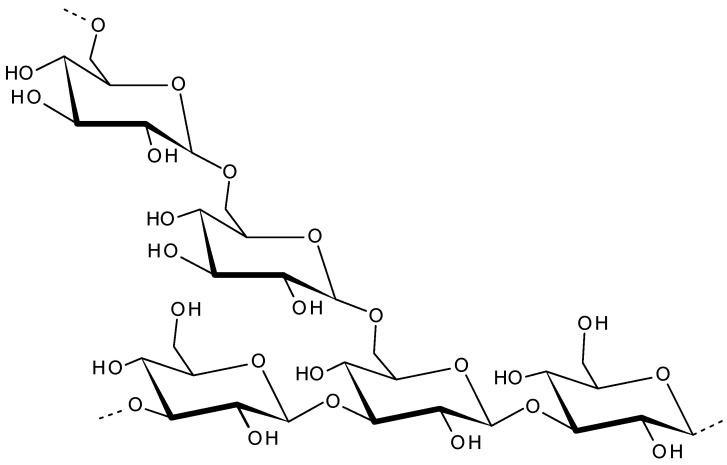
Laminaran structure repeating units.

The main storage polysaccharide of Laminaria species brown algae is called laminaran (also called laminarin). Being a food reserve of brown algae, in cells laminaran is located inside vacuoles. The major sources of laminaran are *Laminaria hyperborean*, *Laminaria digitata*, *Fucus vesiculosus*, *Ascophyllum nodosum*, *Saccharina latissimi*, and *Saccharina longicruris*. Being a low-molecular weight storage β-glucan, Laminaran is composed of (1,3)-β-D-glucan and some β-(1,6)-intrachain links [140] as shown in Figure 8. Laminaran content in brown algae is up to 35% of its dry weight [5,141]. Laminaran is used for cosmetic, food and therapeutic applications [142]. Laminaran is utilized as a water soluble dietary fibre [143]. The dietary supplementation with laminaran in Wistar rat models enhanced the immune response in the hepatic tissue and prevented hepatotoxicity [144]. Laminaran from *Laminaria digitate* found to be efficient in improving the gut health in pigs [145]. When evaluated its effects on intestinal parameters of the gut microflora through the monitoring of biochemical and microbiological parameters, laminaran found to be a modulator of the intestinal metabolism by its effects on mucus composition, intestinal pH and short chain fatty acid production [146]. Additionally, Laminaran is utilized as a functional ingredient in pork meat to improve the shelf life [147,148,149]. Laminaran is a biocompatble polymer and has distinct therapeutic functions. It has excellent antioxidant, antibacterial anti-inflammatory, anti-apoptotic, anti-tumor, antioxidant anticoagulant, immunoregulatory and wound healing properties [150,151,152,153].

The anti-oxidative and anti-inflammatory properties of Laminaran is tested on human dermal fibroblasts adult and normal human epidermal keratinocytes. The mitochondrial activities various cytokine interactions were evaluated, from the results laminaran exhibited a protective effect to regualte the oxidtive stress induced by H_2_O_2_ and UVA radiation and modulates the cytokines and thus control the inflammation [154]. The antitumour acivites of modified and unmodifiied laminaran is tested to check the effect of sulfate modifcation on structure and antitumor activity. The in vitro cytotoxicity evaluation on human colon LoVo cells showed higher inhibitory effects for sulfated laminaran on growth, and the antitumor activity [155]. In gene therapy for breast cancer treatement, laminaran is utilized as a suitable nanovector for the siRNA transfer to downregualte the gene expression. The cationic PEI-modified laminaran showed excellent transfection efficiency (95.0%) in siRNA delivery to MCF-7 cells. This targeted therapy resulted in 90.9% reduction of the gene expression. The in vivo evaluation of the laminaran vector exhibited low toxicity, good gene-therapeutic efficacy, with a tumor inhibition rate of 46.6% [156]. 

Laminaran from *Laminaria digitata*, has anti-inflammatory and anti-oxidative properties which is utilized in the skin cell-based applications including cosmetics and wound healing. The influence of laminaran on skin cells’ (human dermal fibroblasts adult and normal human epidermal keratinocytes) mitochondrial activities are investigated for 72 h. The oxidative stress created by exposure to H_2_O_2_ and UVA radiation conditions, an antioxidant effect was found against reactive oxygen species by the laminaran. Moreover, laminaran treatment controlled the cells surface glycosylation and cytokine secretions of skin cells [154]. 

Laminaran functionalized with phenylboronic acid were used for the synthesis of hydrogels suitable for tissue engineering and drug delivery applications. These reactive oxygen species-responsive hydrogels exhibited enhanced rheological properties and a rapid self-healing behavior upon rupture. The presence of boronate ester bonds supported the fabrication of antioxidant, shear-thinning gels that can be administered to the surrounding microenvironment. When treated with preosteoblasts for up to 48 h, the laminaran hydrogels did not exhibit any cytotoxicity responses [157]. 

To evaluate the effect of Laminaran on glutamic oxaloacetic transaminase and glutamic pyruvic transaminase levels in vivo studies were performed in BALB/c mice models induced with leukemia. When included in diet, laminaran increased the body weight but did not influence the level of marker Mac-3. For different doses, laminaran showed increased NK cell cytotoxic activity and did not affect B-cell proliferation, but at 5 mg/mL it reduced T-cell proliferation. Laminaran also restored glutamate oxaloacetate transaminase and glutamate pyruvate transaminase levels. These results supported the immune responses and liver protection potential of laminaran in in vivo models [158]. 

Laminaran when tested on hamster models exhibited inhibiting effect of liver metastasis of pancreatic cancer. Sodium laminaran is introduced to the test animals a dosage dependent inhibition was observed towards the growth, metastasis and angiogenesis of pancreatic cancer, and explore its mechanism [159]. Similarly, in presence of superoxide dismutase and malondialdehyde, introduction of Laminaran showed brain tissue protection via anti-lipid peroxidation [160]. The introduction of Laminaran to human colon cancer HT-29 cells utilized to analyze its effect on the insulin-like growth factor signaling pathway. From the results, laminaran decreased mitogen-activated protein kinases (MAPK) and ERK phosphorylation. Moreover, a dose-dependent cell death is observed with Laminaran introduction which could be due to the activation of Fas-induced apoptosis [161]. 

The immunostimulatory effects of laminaran were tested on mouse macrophages RAW 264.7 cells. Laminaran significantly improved the release of calcium, hydrogen peroxide, nitric oxide, monocyte chemotactic protein-1, vascular endothelial growth factor, leukemia inhibitory factor, and granulocyte-colony stimulating factor with enhancing expression of Signal Transducer and Activator of Transcription 1 (STAT1), STAT3, c-Jun, c-Fos, and cyclooxygenase-2 mRNA in RAW 264.7 cell. Results showed the immunostimulatory properties of laminaran in strengthening immune reactions through the transcription factor pathway in macrophages [162]. 

Laminaran promoted the defense response in grapevine towards protection from plant pathogens like *Botrytis cinerea* and *Plasmopara viticola.* The defense reactions include calcium influx, extracellular medium alkalinization, an oxidative burst, activation of mitogen-activated protein kinases, defense-related genes’ expression, enhancement in chitinase and β-1,3-glucanase activities, phytoalexins production. Laminaran did not cause cell death, but helped in reducing the infection significantly [163]. Similarly, laminaran is found efficient against Tobacco mosaic virus (TMV). When the tobacco leaves were treated with laminaran, they displayed systemic resistance and protection effect on host against the virus [164]. Table 1includes more antiviral applications of sulfated marine polysaccharides [136,165,166,167,168,169,170,171,172,173,174,175,176,177,178]. 

## 3. Conclusions

Marine polysaccharides are structurally diverse and heterogeneous high-value bioproducts with numerous beneficial functional properties. The antiviral mechanisms of these polysaccharides depend on multiple factors such as degree of sulfation, molecular weight, composition, and structure, etc. A broad range of antiviral mechanisms, low production costs, low cytotoxicity, and wide acceptability of marine algae polysaccharides could lead to promising antiviral drugs. Furthermore, the immunomodulatory effect of marine polymers to develop vaccines and generate innate immunity in organisms to prevent infections is another interesting area to be explored further. The cytocompatibility of these biomolecules to normal cells enable the development of various advanced nanobiomaterials for tissue engineering and regenerative medicine. The antiviral properties of marine polysaccharides and its synergistic effects with other antiviral agents showed remarkable antiviral responses for upcoming medical explorations. The significant inhibitory activities of marine polysaccharides against SARS-CoV-2 are a remarkable achievement for the development of future algal therapeutics and research. The majority of the pharmacological studies of these polysaccharides were limited by pre-clinical (in vitro and in vivo) evaluations, where clinical responses are pivotal for the development of pharmaceutical drugs. Once clinically approved, being biogenic, biocompatible and renewable, the marine algae polysaccharide based antiviral products will have a high demand in the upcoming pharmaceutical industry.

## Figures and Tables

**Table 1:** Recent antiviral investigations of various sulfated marine polysaccharides.

Seaweed Name (Source)	Polysaccharide Name	Activity (Targets)	References
*Solieria filiformis*	carrageenan	in vitro cytotoxicity and antiviral activity against HSV-1	[165]
Not mentioned	lambda-carrageenan	in vitro and in vivo antiviral activity influenza A and B viruses and SARS-CoV-2	[166]
*Sargassum ilicifolium*	sulfated polysaccharides	in vitro and in vivo antiviral activity against fish betanodavirus	[167]
*Sargassum fusiforme*	sargassum fusiforme polysaccharide (SFP-1, SFP-2, SFP-3, SFP-4 and SFP-5)	in vivo and in vitro antiviral activity-avian leukosis virus, Subgroup J	[168]
*Monostroma nitidum*	homogeneous polysaccharide (MWS)	antiviral activity against influenza virus, HSV and EV71	[169]
*Sargassum henslowianum*	fucoidans SHAP-1 and SHAP-2	in vitro antiviral activity against HSV-1	[170]
red seaweed	carrageenan	in vitro and in vivo antiviral activity against Japanese encephalitis virus (JEV)	[171]
*Ulva pertusa*	ulvan	antiviral activity against vesicular stomatitis virus	[136]
*Laminaria japonica*	fucose, galactose, and mannose	in vitro inhibition of enterovirus EV71 and 3C protein activity	[172]
*Solieria filiformis*	polyphenol-rich extracts	antiviral activity against measles virus (MeV)	[173]
*Gracilaria birdiae*	sulfated polysaccharides	white spot syndrome virus (WSSV)	[174]
*Grateloupia filicinia*	polysaccharides	Antiviral activity towards avian leucosis virus (ALV-J)	[175]
*Ulva intestinalis*	crude extract	antiviral activity against white spot syndrome virus (WSSV) and yellowhead virus (YHV)	[176]
*Monostroma nitidum*	rhamnan sulfate	anti-influenza A virus activity	[177]
*Macrocystis pyrifera* *Durvillaea antarctica*	extract	anti-herpetic activity against HSV-1 and HSV-2	[178]
